# Identification of Genes Linked to Meniscal Degeneration in Osteoarthritis: An In Silico Analysis

**DOI:** 10.3390/ijms26146651

**Published:** 2025-07-11

**Authors:** Aliki-Alexandra Papageorgiou, Charalampos Balis, Ioanna Papathanasiou

**Affiliations:** Laboratory of Cytogenetics and Molecular Genetics, Faculty of Medicine, School of Health Sciences, University of Thessaly, Biopolis, 41500 Larissa, Greece; alipapageorgiou@med.uth.gr (A.-A.P.); chbalis@uth.gr (C.B.)

**Keywords:** osteoarthritis, meniscus, transcriptome, in silico analysis

## Abstract

Meniscal degradation is considered a driver of osteoarthritis (OA) progression, but the underlying mechanisms leading to age-related meniscus degeneration remain unknown. This study aimed to identify key genes and pathways involved in meniscal degradation through a computational analysis. Gene expression profiles were obtained from the Gene Expression Omnibus (GEO) database. Differential expression gene (DEG) analysis was performed using DESeq2 accompanied by functional enrichment analysis, protein–protein interaction (PPI) and clustering analysis. Additionally, gene set enrichment analysis (GSEA) was performed. A total of 85 mRNAs (DEMs) and 8 long non-coding RNAs (DE LncRNAs) were found to be differentially expressed in OA meniscus tissues. Among 85 DEMs, 12 genes were found to be known OA-related genes, whereas 15 genes acted as transcription regulators, including *RUNX2* and *TBX4*, which were identified as effector genes for OA. Enrichment analysis revealed the implication of DEMs in cartilage-degradation-related processes, including inflammatory pathways, lipid metabolism, extracellular matrix organization and superoxide/nitric oxide metabolic processes. Target genes of DE lncRNAs were found to be involved in chondrocyte differentiation and pathways related to cartilage degradation. A comparative analysis of meniscus, synovium and cartilage datasets identified three genes (*GJB2*, *PAQR5* and *CLEC12A*) as being differentially expressed across all three OA-affected tissues, which were implicated in inflammatory and cholesterol metabolism processes. Our results support that shared mechanisms lead to meniscal and cartilage degradation during OA progression, providing further insights into the processes underlying OA pathogenesis and potential therapeutic targets for knee OA.

## 1. Introduction

Osteoarthritis (OA) is the most prevalent, chronic and disabling form of arthritis affecting almost 500 million people worldwide [1]. OA is characterized as a whole-joint disease that encompasses various histological and structural changes across all joint tissues, including cartilage degradation, synovial membrane inflammation, subchondral bone thickening and sclerosis, infrapatellar fat pad fibrosis and inflammation, meniscus fractures and ligament damage, ultimately leading to whole-joint dysfunction [2,3,4]. Currently, no curative treatments are available, and management strategies focus on alleviating symptoms through pain relief and arthroplasty [3,4]. Thus, a more comprehensive understanding of OA as a disease of the entire joint is essential for the development of effective treatment strategies in the early stages of OA.

Although the destruction of articular cartilage is the hallmark of the majority of knee OA, synovial inflammation is also considered a common feature of OA jointly contributing to both pain and disease development [5,6]. Moreover, the meniscus, a fibrocartilaginous tissue, plays a vital role in knee biomechanics by acting as a component in maintaining joint stability and shielding against excessive stress [7]. The loss of meniscal function due to traumatic (acute) or degenerative (chronic) damage contributes to the development and progression of primary OA through several mechanisms, including the release of pro-inflammatory cytokines and increased bone remodeling (osteophyte formation) [8]. Meniscal tears or extrusion can evoke joint space narrowing, amplify cartilage deterioration and further disrupt load distribution and shock absorption [9,10]. Furthermore, meniscal tears in middle-aged and elderly patients often precede radiographic knee OA, suggesting that meniscus pathology may be considered an early indicator/driver of OA [11]. However, the molecular mechanisms underlying meniscal degeneration during OA are not yet fully understood.

Given that OA is a multifactorial disease driven by a combination of genetic and epigenetic factors, its pathological progression is intricately linked to changes in multiple genes, as well as epigenetic mechanisms, including the function of long non-coding RNAs (lncRNAs) [12,13]. LncRNAs have emerged as crucial regulators in the OA pathogenesis by regulating the key molecular mechanisms involved in cartilage degradation, inflammation and extracellular matrix (ECM) remodeling [14,15,16]. Several lncRNAs such as H19, XIST, MALAT1 and NEAT1 modulate chondrocyte apoptosis, proliferation and ECM degradation/synthesis through interactions with miRNAs and signaling pathways including NF-κB and TGF-β/SMAD [13,16,17]. These regulatory mechanisms have also been implicated in meniscal pathology, as meniscal degeneration is primarily driven by mechanical stress and inflammation. Previous studies reported that dysregulated lncRNAs contribute to meniscal damage by altering gene expression patterns and promoting inflammatory cascades [14]. Understanding the role of lncRNAs in the degenerative processes of meniscus provides promising avenues for developing novel biomarkers and targeted therapies for OA patients [13,15,18].

Recent advances in high-throughput transcriptomic technologies and computational biology have contributed to exploring the complex molecular landscape in OA [19,20]. Regarding degenerative meniscus, whole- and single-cell transcriptomic analysis revealed distinct phenotypes and different expression patterns among normal, injured and OA meniscus which were associated with OA-related processes including inflammation, apoptosis and autophagy, giving insights into how meniscus gene expression relates to OA onset and progression [21,22,23,24]. More importantly, by comparing differential gene expression profiles of different joint tissues, including articular cartilage, synovium and meniscus, the current research aims to identify overlapping differentially expressed genes (DEGs) and, subsequently, the disrupted biological pathways [23,25,26] that lead to whole-joint dysfunction, highlighting specific biomarkers at the RNA level for diagnosing early-stage OA and targets for new therapeutic strategies.

In this study, we aimed to identify the key genes associated with degenerative changes in the meniscus in OA, performing an in silico analysis of a publicly available gene expression dataset to provide insights into OA pathophysiology for developing future diagnostic and therapeutic interventions.

## 2. Results

### 2.1. mRNA Expression Profile in OA Meniscus

**Figure 1:**
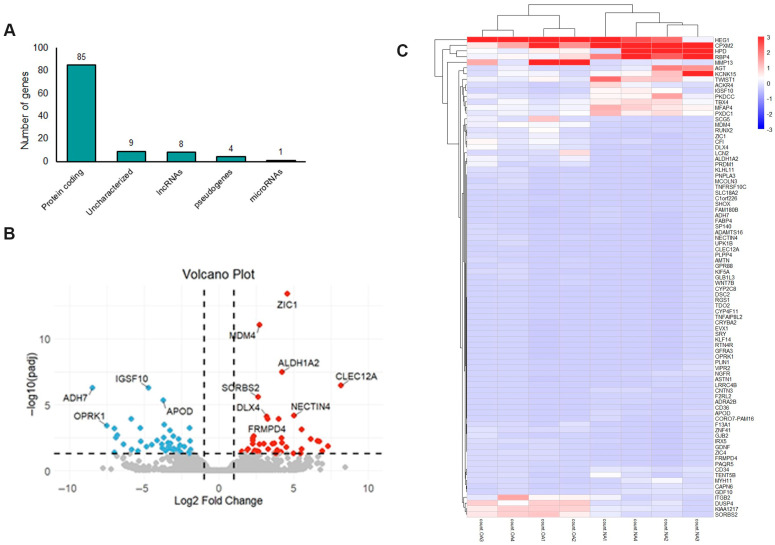
mRNA expression profile of OA meniscus: (**A**) Different biotypes of genes differentially expressed between OA and healthy meniscus (adjusted *p*-value < 0.05). (**B**) Volcano plot showing the differentially expressed mRNAs between OA and healthy meniscus (adjusted *p*-value < 0.05; absolute log2 fold change > 1). Red dots indicate significantly upregulated mRNAs and blue dots significantly downregulated mRNAs. Gray dots represent mRNAs without significant differential expression. (**C**) Heatmap indicating the expression patterns of OA and healthy meniscus.

For the GSE185064 dataset, differential gene expression analysis was performed using DESeq2 to determine the genes that were differentially expressed in the OA meniscus compared to the healthy ones. Compared to the healthy meniscus, 107 transcripts were significantly differentially expressed (adjusted *p*-value < 0.05) in the OA meniscus, representing different biotypes, e.g., protein coding (85 transcripts), lncRNAs (8 transcripts), pseudogenes (4 transcripts) and uncharacterized genes (9 transcripts). Only miR-1244-1 was found to be significantly elevated in OA meniscus compared to healthy meniscus (log2FC = 0.954) (Figure 1A).

As presented in the volcano plot (Figure 1B), 85 mRNAs were differentially expressed (DEMs) between the OA and healthy meniscus, with 41 being downregulated and 44 upregulated in OA meniscus (Table S1). C-Type Lectin Domain Family 12 Member A (*CLEC12A*) gene (log2FC = 8.16), which encodes a myeloid inhibitory receptor associated with the pathogenesis of rheumatoid arthritis (RA) [27], was the most upregulated in the OA meniscus. Moreover, matrix metalloproteinase 13 (*MMP13*) (log2FC = 3.59) and ADAM metallopeptidase with thrombospondin type 1 motif 16 (*ADAMTS16*) (log2FC = 4.18) were found to be overexpressed in the OA meniscus compared to the healthy ones. Among the most downregulated genes were the following: Alcohol Dehydrogenase 7 (*ADH7*) (log2FC = −8.46), Opioid Receptor Kappa 1 (*ORPK1*) (log2FC = −7.50), KLF Transcription Factor 14 (*KLF14*) (log2FC = −6.80) and CORO7-PAM16 Read through (*CORO7-PAM16*) (log2FC = −4.84). A heatmap visualizing the expression pattern of differentially expressed mRNAs in the OA meniscus is shown in Figure 1C.

### 2.2. Functional Enrichment Analysis of Differentially Expressed mRNAs (DEMs) in OA Meniscus

To further understand the biological functions of differentially expressed mRNAs (DEMs) in meniscal degeneration and, subsequently, OA progression, functional enrichment analyses based on Gene Ontology (GO) were performed. Utilizing the DAVID database, GO Biological Process (GO-BP) analysis revealed that the DEMs were primarily associated with retinol metabolic process, ossification, skeletal system development and bone mineralization; GO Cell Component (GO-CC) analysis demonstrated that they were mainly located in extracellular region, in specific granule membranes or plasma membranes and GO Molecular Function (GO-MF) analysis showed that they linked with DNA-binding transcription activator activity and cytoskeletal motor activity (Table 1). Additionally, enrichment analysis based on KEGG and Reactome pathway databases indicated that the DEMs were involved in retinol metabolism, PPAR signaling, interleukin-4 and interleukin-13 signaling, metabolism of lipids and extracellular matrix organization (Table 1). CD36 molecule (*CD36*) and Integrin Subunit Beta 2 (*ITGB2*) genes were most enriched in these identified pathways.

**Figure 2:**
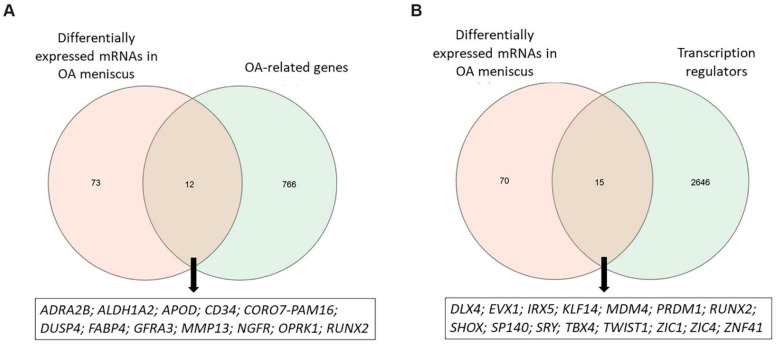
Identification of OA-related DEMs and transcription regulators. (**A**) Venn diagram showing the overlap between differentially expressed mRNAs observed in OA meniscus and OA-related genes obtained by Open Targets platform. (**B**) Venn diagram showing the overlap between differentially expressed mRNAs observed in OA meniscus and transcription regulators obtained by the AnimalTFDB database.

By intersecting the genes associated with OA pathogenesis retrieved by Open Targets platform with 85 DEMs, as identified by performing differential expression analysis, 12 DEMs were found to be known OA-related genes, including CD34 molecule (*CD34*), *MMP13*, Apolipoprotein D (*APOD*), *OPRK1* and RUNX Family Transcription Factor 2 (*RUNX2*) (Figure 2A). Moreover, we found that 20% of genes that were differentially expressed in OA meniscus acted as transcriptional regulators (TFs or cofactors) of gene expression (Figure 2B). Among them, *RUNX2* and T-Box Transcription Factor 4 (*TBX4*) are major regulators of chondrogenesis and have previously been identified as effector genes for osteoarthritis [28,29,30]. Twist Family BHLH Transcription Factor 1 (*TWIST1*) gene encodes a transcriptional factor that controls *COL2A1* and *SOX9* gene expression in chondrocytes, whereas SHOX Homeobox (*SHOX*) regulates the collagen type I and MMP expression in articular cartilage [31,32].

### 2.3. Protein–Protein Interactions of Differentially Expressed mRNAs (DEMs) in OA Meniscus

**Figure 3:**
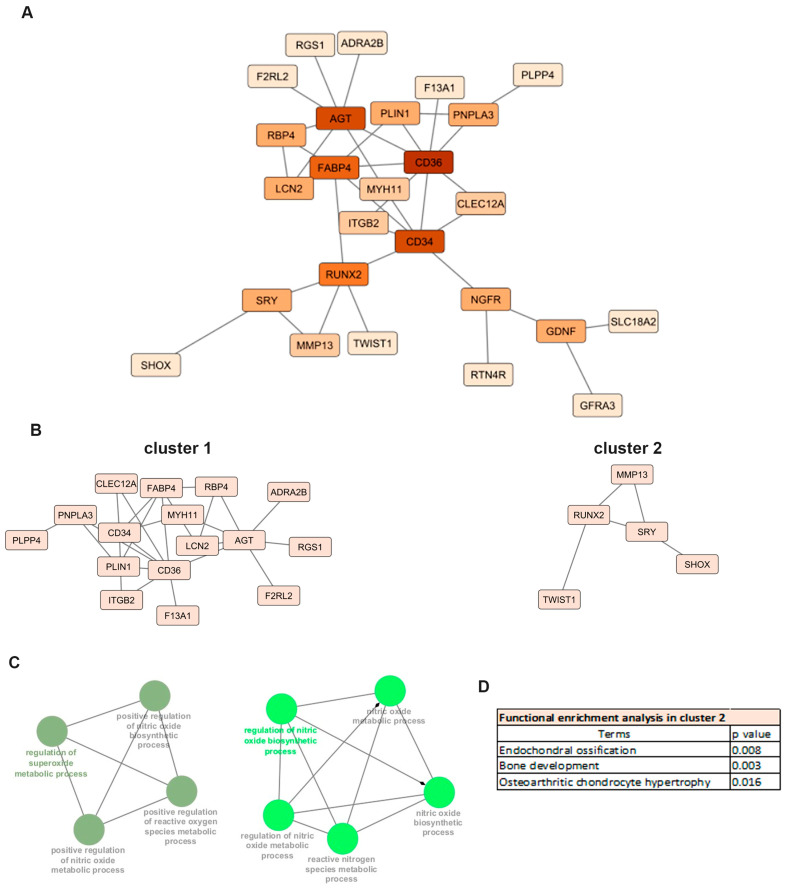
Protein–protein interaction (PPI) analysis: (**A**) PPI network of differentially expressed mRNAs in OA meniscus. The nodes represent the target genes and the edges indicate both functional and physical protein associations. A high confidence score of 0.400 was defined as the minimum interaction score to construct the PPI network. (**B**) Sub-networks after MCL clustering. (**C**) Enrichment analyses of cluster 1 using the ClueGo plugin of Cytoscape. (**D**) Functional enrichment analysis of cluster 2 using g:Profiler.

Functional interactions of proteins encoded by the 85 differentially expressed mRNAs (DEMs) in OA meniscus were investigated by protein–protein interaction (PPI) analysis. The main constructed network comprised 26 nodes with 36 edges. Among 26 nodes, 5 nodes represent genes with higher connectivity (degree (k) ≥ 5) and potential regulatory importance within the network, suggesting their central role in meniscus degradation during OA progression (Figure 3A). Among them, the CD34 molecule (*CD34*), *RUNX2* and Fatty Acid Binding Protein 4 (*FABP4*) genes are known OA-related genes, as predicted by the Open Target platform.

MCL clustering of the PPI network revealed two main clusters consisting of 16 nodes with 24 edges (cluster 1) and 5 nodes with 5 edges (cluster 2), respectively (Figure 3B). Genes located in cluster 1, including *CD36*, *CD34*, Angiotensinogen 2 (*AGT*), *FABP4* as well as *CLEC12A*, exhibited the highest expression in the OA meniscus and were mainly enriched in biological processes related to cartilage degradation such as superoxide and nitric oxide metabolic processes (Figure 3C). OA-related genes *RUNX2* and *MMP13*, the major catabolic factor of articular cartilage, were defined as a part of cluster 2 and enrichment analysis revealed their implication in endochondral ossification and osteoarthritic chondrocyte hypertrophy (Figure 3D). All the above results support that common biological processes are disrupted in multiple joint components leading to OA onset and progression.

### 2.4. Common Transcriptional Changes in Meniscus, Synovium and Cartilage in OA

**Figure 4:**
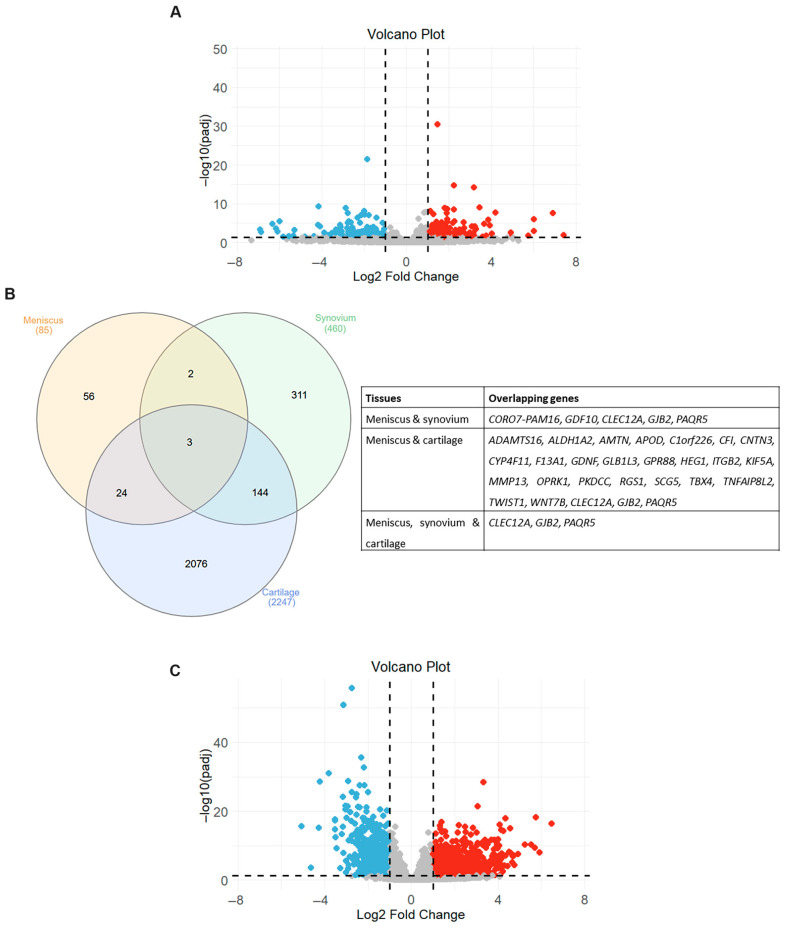
Overlapping differentially expressed genes (DEGs) in different joint components. (**A**) Volcano plot showing differentially expressed mRNAs between OA and healthy synovium samples. Red dots indicate significantly upregulated mRNAs and blue dots significantly downregulated mRNAs (adjusted *p*-value < 0.05 and |log2 fold change| > 1). Gray dots represent mRNAs without significant differential expression. (**B**) Venn diagram and table showing the overlapping DEGs in three OA-affected joint tissues. (**C**) Volcano plot showing differentially expressed mRNAs between OA and healthy cartilage samples. Red dots indicate significantly upregulated mRNAs and blue dots significantly downregulated mRNAs (adjusted *p*-value < 0.05 and |log2 fold change| > 1). Gray dots represent mRNAs without significant differential expression.

We next proceeded to investigate whether the transcriptional changes observed in the meniscus of OA patients are consistent with transcriptomic profiles from other affected components of the OA joints, including synovium and cartilage. The differentially expressed genes in synovium and cartilage were identified using the DESeq2 package based on the GSE143514 and GSE114007 datasets, respectively. For the GSE143514 database, 460 genes were found to be significantly differentially expressed (padj < 0.05 and absolute log2FC > 1) in synovial tissues from OA patients, of which 281 were upregulated and 179 were downregulated (Table S2). The volcano plot visualizing the expression pattern of differentially expressed mRNAs in OA synovium is shown in Figure 4A. Of the overlapping differentially expressed genes from meniscus and synovial tissues, only five genes were found to be differentially expressed in both OA tissues (Figure 4B). Gap Junction Protein Beta 2 (*GJB2*), Progestin And AdipoQ Receptor Family Member 5 (*PAQR5*) and *CLEC12A* were upregulated in the OA synovium and meniscus, whereas *CORO7-PAM16* and Growth Differentiation Factor 10 (*GDF10*) were upregulated in the synovium and downregulated in the meniscus from OA patients (Table 2).

After differential gene expression analysis of the GSE114007 database, 2247 genes were considered significantly differentially expressed (padj < 0.05 and absolute log2FC > 1) in OA cartilage samples. More specifically, we found that 1311 genes were significantly upregulated and 936 genes downregulated in OA cartilage tissues compared to normal ones (Table S3). The volcano plot visualizing the expression pattern of differentially expressed mRNAs in OA cartilage is shown in Figure 4C. Venn diagram analysis revealed that 32% of genes that exhibited differential expression in OA meniscus also had a disrupted expression in OA cartilage tissues, suggesting that the transcriptional changes observed in the meniscus in an OA joint are more closely related to those of cartilage tissues rather than synovium (Figure 4B).

Among them, 17 genes were upregulated and 4 genes were downregulated in both OA tissues, whereas 6 genes exhibited opposite expression patterns between the OA meniscus and cartilage (Table 3).

**Figure 5:**
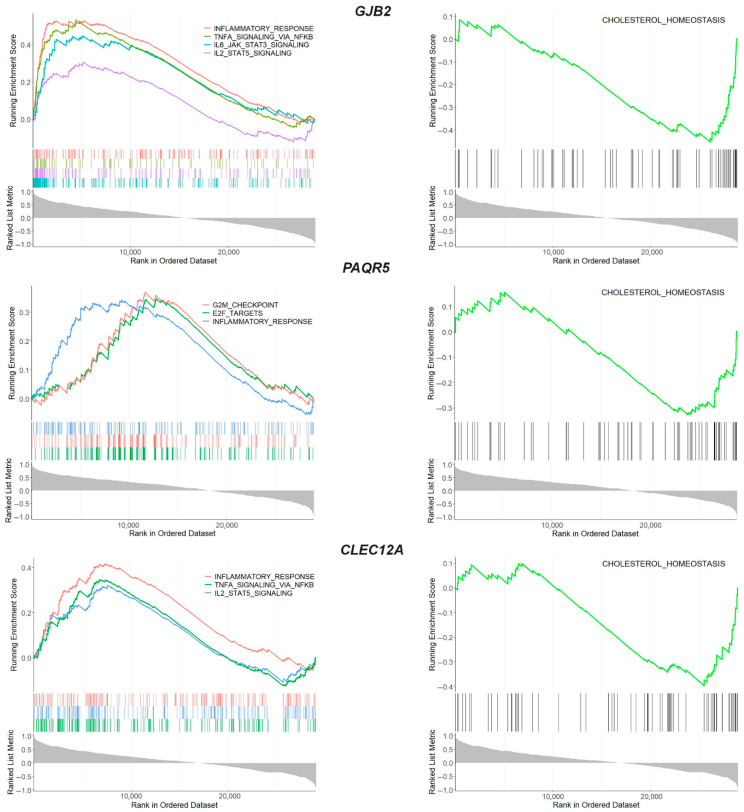
GSEA enrichment analysis of overlapping genes *GJB2*, *PAQR5* and *CLEC12A*. GSEA enrichment plot showing the biological processes in which the overlapping genes were involved.

Comparative analysis of the transcriptional profiles of the three databases revealed that three genes exhibited aberrant expression in the integral joint tissues (Figure 4A). A notable observation was that all of the overlapping genes (*GJB2*, *PAQR5* and *CLEC12A*) showed a similar expression pattern in OA tissues, exhibiting upregulation in the meniscus, synovium and cartilage from OA patients compared to normal tissues. GSEA using the MSigDB database demonstrated that the *GJB2*, *PAQR5* and *CLEC12A* genes activate inflammatory-related signaling (NES > 0) and suppress the cholesterol homeostasis (NES < 0), revealing their role in the meniscal degradation process during osteoarthritis progression (Figure 5 and Tables S5–S7).

### 2.5. Long Non-Coding RNA (lncRNA) Profile in OA Meniscus

**Figure 6:**
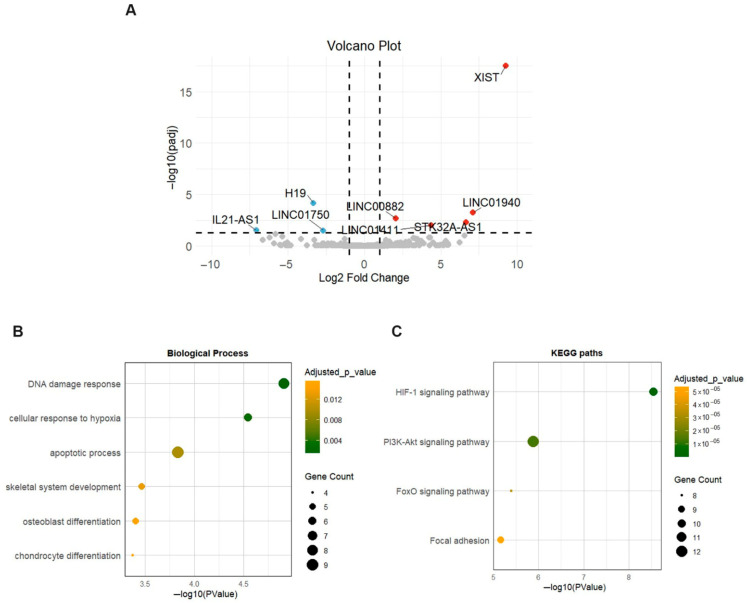
Expression profile of lncRNAs in OA meniscus. (**A**) Volcano plot showing differentially expressed lncRNAs between OA and healthy meniscus samples. Red dots indicate significantly upregulated lncRNAs and blue dots significantly downregulated lncRNAs (adjusted *p*-value < 0.05 and |log2 fold change| > 1). Gray dots represent lncRNAs without significant differential expression. (**B**,**C**) Dot plots show the enriched biological processes and KEGG pathways of target genes of differentially expressed lncRNAs. Dot size indicates the number of targets enriched in each pathway and the dot color reflects the adjusted *p*-value.

Eight lncRNAs were found to be differentially expressed between OA and healthy meniscus, with three being downregulated and five upregulated in OA meniscus (Table S4). X Inactive Specific Transcript (*XIST*) gene (log2FC = 9.24) was the most upregulated in OA meniscus, whereas the downregulated lncRNAs were as follows: IL21-AS (log2FC = −7.06), H19 (log2FC = −3.35) and LINC01750 (log2FC = −2.69). The volcano plot visualizing the expression pattern of differentially expressed lncRNAs (DE lncRNAs) in OA meniscus is shown in Figure 6A.

To gain further insights into the biological functions of DE lncRNAs during the degenerative process in meniscus, mRNA targets of DE lncRNAs were assigned based on the lncRNA2target database. Two of the eight DE lncRNAs, XIST and H19, were identified to target 56 proteins, while six DE lncRNAs had no experimentally validated mRNA targets. Enrichment analysis revealed that targets of DE lncRNAs were mainly implicated in skeletal development and osteoblast and chondrocyte differentiation (Figure 6B). Additionally, KEGG enrichment analysis indicated that they were associated with OA-related pathways including the HIF-1 signaling pathway, PI3K-Akt signaling pathway, FoxO signaling pathway and Focal adhesion (Figure 6C).

### 2.6. Drug Targets

The Open Targets platform was used to identify which of the key genes (*CD34*, *CD36*, *AGT*, *FABP4*, *RUNX2*, *ITGB2* and *MMP13*) implicated in meniscal degradation, as predicted by enrichment and PPI analysis, could be targeted by known drugs serving as potential targets for intra-articular intervention. We found that only *MMP13* and *ITGB2* express a protein that is a target of approved drugs (doxycycline and lifitegrast, respectively). Moreover, no known drugs are available against the three overlapping genes (*GJB2*, *PAQR5* and *CLEC12A*), lncRNA XIST and lncRNA H19.

## 3. Discussion

Emerging evidence suggests that age-related meniscal degeneration is closely associated with the onset and progression of OA [9]. Advances in transcriptomic technologies, such as RNA sequencing, have enabled the identification of differentially expressed genes and dysregulated signaling pathways involved in complex diseases, such as OA [33,34]. Despite these advances, and the observation of distinct gene expression alterations in degenerative meniscus tissue, the underlying mechanisms that drive meniscal deterioration during osteoarthritis development are still not fully understood.

To understand the transcriptional signature of degenerative meniscus, we performed differential gene expression analysis on a previously published knee osteoarthritis (OA) RNA-seq dataset (GSE185064) and identified 85 mRNAs to be differentially expressed between OA and healthy meniscus. More specifically, compared to healthy meniscus, the OA meniscus elevated its catabolic action by increasing the expression levels of catabolic enzymes such as ADAMTS-16 and MMP-13. In agreement with our results, ADAMTS-16 expression was also found to be increased in OA cartilage and synovium [35,36]. Although overexpression of ADAMTS-16 led to decreased MMP-13 expression in chondrosarcoma cells, the role of ADAMTS-16 in OA progression and especially in the meniscal degradation is still unknown [37]. Regarding MMP-13 expression in OA meniscus, previous studies, consistent with our findings, demonstrated the increased expression of MMP-13 in the meniscus of OA patients as well as a link between MMP-13 expression in patients with meniscal tears and pain [38,39]. Moreover, in vivo studies indicated the increased expression of MMP-13 in degenerated meniscus in OA animal models, which was possibly associated with meniscal tear formation that contributed to OA development [40,41]. MMP-13 is a well-known driver of cartilage degradation due to its capacity to degrade extracellular matrix components, including collagen type II and aggrecan, producing products that can stimulate synoviocytes to release inflammatory factors and MMPs and enhancing the degradative processes in all joint tissues [42]. Moreover, increased expression of MMP-13 is associated with the hypertrophic phenotype of chondrocytes that lead to greater cartilage damage and OA progression [43]. All the above support the crucial role of the disrupted expression of MMP-13 in the extracellular matrix degradation of both articular cartilage and meniscus, highlighting MMP-13 as a potential therapeutic target for managing OA progression and improving joint health.

Besides increased catabolic enzymes, differential gene expression analysis revealed that OA meniscus had elevated expression levels of ITGB2, a member of the family called beta2 integrins that are crucial for cell adhesion, cell communication and signal transduction [44]. Enrichment analysis revealed that the *ITGB2* gene is enriched in processes that are disrupted during OA progression, such as extracellular matrix organization and inflammation. A previous study, in line with our results, revealed that ITGB2 expression was remarkably upregulated in OA meniscal cells compared to that in normal meniscal cells [45]. Moreover, compared to normal joint tissues, increased ITGB2 expression levels were also observed in OA cartilage and bone tissues as well as in the synovial fluid of OA patients, which was correlated with disease severity [46,47]. Recently, the *ITGB2* rs2070946 single nucleotide polymorphism has been associated with increased susceptibility to OA, suggesting a genetic predisposition linked to integrin-mediated pathways [48]. In addition, β2 integrins exist widely in human immune cells and their disrupted expression seems to contribute to the synovitis development observed in an OA joint [49]. The abnormal expression of ITGB2 gene in all OA-affected tissues as well as the strong link of β2 integrins with OA-related processes, including cartilage degradation, bone remodeling and low-grade inflammation, makes β2 integrins candidate targets for OA treatment.

During OA progression, it is well known that chondrocytes acquire a hypertrophic phenotype which is characterized by the increased activity of degradative enzymes, expression of hypertrophic markers such as collagen type X (COLX) and alkaline phosphatase (ALP), but also the disrupted expression of transcription factors implicated in chondrogenesis, including SOX9 and RUNX2 [50]. By intersecting the transcriptional regulators from the AnimalTFBD4 database with the 85 differentially expressed genes, 15 transcriptional regulatory genes were identified. Among them, the *RUNX2* and *TBX4* genes, known to regulate the growth of skeletal elements, exhibited abnormal expression in OA meniscus. *RUNX2* is an OA-related gene that normally triggers the chondrocyte terminal differentiation and endochondral ossification [51,52] and is found to be upregulated in OA meniscus. Experimental findings have displayed the catabolic action of RUNX2 in adult articular cartilage, as the over-activation of RUNX2 drives the transition of adult quiescent chondrocytes to the hypertrophic phenotype by inducing the expression of hypertrophic markers, leading to cartilage degradation and subsequently OA onset and progression [53,54]. Besides RUNX2, transcription factor TBX4 has been previously identified as an effector gene for OA development [55]. Our results showed a decreased expression of TBX4 in OA meniscus compared to healthy meniscus. Previously, Alvarez-Garcia et al. demonstrated that transcription factor TBX4 was significantly hypermethylated and downregulated in OA cartilage [56]. More recently, TBX4 has been identified as a central transcriptional regulator of cartilage degeneration in knee osteoarthritis, having important roles in chondrogenesis and embryonic limb morphogenesis [57]. Overall, these results propose that the disrupted expression of specific transcription factors related to developmental processes such as chondrogenesis contribute to degenerative changes observed in OA-affected tissues such as meniscus and articular cartilage.

After performing MCL clustering in the PPI network of differentially expressed genes, enrichment analysis revealed the involvement of superoxide and nitric oxide metabolic processes in the degenerative processes of meniscus, which have been identified as master contributors to OA development and progression [58,59], since expression modifications of genes implicated in these pathways were observed in affected joint tissues [58,60]. The diminished activity of superoxide dismutase (SOD) enzymes in OA-damaged cartilage [58,61] disrupts the redox equilibrium in chondrocytes, causing mitochondrial dysfunction, heightened ROS generation and eventual cartilage breakdown. Inflammatory conditions worsen this cycle by inducing synovitis and hypoxia in synovial cells, further elevating ROS levels [58]. Regarding the link between NO and OA, the production of NO is driven by the inducible nitric oxide synthase (iNOS) enzyme, which becomes more active in chondrocytes and synovial cells when exposed to inflammatory signals [59,62]. This upregulation results in an excessive release of NO that accelerates cartilage breakdown by inducing the activity of matrix metalloproteinases, reducing the production of collagen and proteoglycans and triggering chondrocyte apoptosis [59,60]. Similarly, disrupted function of the above metabolic processes may affect the function of the meniscus, leading to aging-related meniscal damage and eventually OA development.

By comparing publicly available datasets on cartilage and synovium with a meniscus dataset, three overlapping genes (*GJB2*, *PAQR5* and *CLEC12A*) that have not been previously associated with OA were found to be differentially expressed across all three OA-affected tissues, following the same direction of change. The *GJB2* gene encodes connexin 26 (Cx26), a member of the gap junction protein family (connexins or GJs) [63]. Differential expression of GJB2 has also been reported in the OA cartilage and synovium by Li et al. [64], but its role in the OA pathogenesis remains unknown. Generally, gap junction (GJ) communications play an important role in cartilage homeostasis, especially after mechanical stress responses, and the altered expression levels of connexins observed in OA may disrupt this communication network, contributing to the progressive destruction of articular cartilage [65,66]. Moreover, inflammatory cytokines released by synovial fibroblasts lead to the pathological expression of connexins in both synoviocytes and chondrocytes, promoting cartilage destruction [67]. The *PAQR5* gene encodes a membrane receptor implicated in progesterone signaling and implicated in inflammatory responses by binding to the TNF-α-responsive region and regulating NADPH oxidase activity [68]. Previous studies reported the involvement of PAQR5 in cancer cell biology [69,70], but its role in musculoskeletal tissues and diseases remains to be further explored. The *CLEC12A* gene encodes a C-type lectin domain-containing receptor with inhibitory functions, known to regulate inflammation and immune responses [71]. While CLEC12A plays an important regulatory role in inflammatory arthritis like RA [27,72], no evidence currently links it to OA. In our study we found that the three overlapping genes were upregulated in all OA-affected tissues, and enrichment analysis revealed their implication in the activation of inflammatory pathways and the inactivation of cholesterol metabolism. It is well known that the tissues in the joint, including the cartilage, meniscus and synovium, interact to contribute to inflammation-associated cartilage degradation, highlighting that inflammation is a key driver of OA development and progression [73]. Regarding cholesterol metabolism, disrupted cholesterol-related mechanisms have been demonstrated to be involved in the development of OA, leading to abnormal lipid accumulation in chondrocytes and, subsequently, their increased catabolic action [74]. Based on the results, we propose three novel genes implicated in hallmark OA-related processes as targets for OA treatment, with benefits in all OA-affected tissues.

Besides distinct transcriptional changes in the mRNA pattern of OA meniscus, eight lncRNAs have been demonstrated to be differentially expressed in the meniscus observed from OA patients compared to healthy ones. Among them, XIST and H19, which are related to cartilage degradation, were found to be upregulated and downregulated in the OA meniscus, respectively. Bioinformatic analysis revealed that genes regulated by XIST and H19 were mainly enriched in skeletal-development-related processes and in pathways implicated in OA pathogenesis, including the HIF-1 signaling pathway, PI3K-Akt signaling pathway and FoxO signaling pathway. Previous studies reported that XIST was consistently upregulated in OA cartilage, contributing to ECM degradation and chondrocyte apoptosis by sponging various miRNAs, including miR-1277-5p, miR-149-5p, miR-142-5p, miR-211 and miR-146a [17,75,76,77,78]. Moreover, increased expression of XIST was also found in both temporomandibular joint osteoarthritis (TMJOA) synovial tissue and inflammatory-stimulated synovial cells, whereas its inhibition promoted the proliferation of synovial cells upon inflammatory stimulation [79]. Knockdown of XIST has been shown to suppress inflammatory responses, reduce ECM degradation and enhance chondrocyte viability in IL-1β-treated models [75,76,77,78]. Furthermore, Xiao et al. indicated that flavonoid compounds with antioxidant and anti-inflammatory properties, like kaempferol, led to decreased XIST expression and the subsequent inhibition of inflammation and extracellular matrix degradation in chondrocytes, suggesting XIST downregulation as a potential therapeutic target for OA and related meniscus injuries [80]. On the other hand, H19 was highly expressed in damaged cartilage and synovial fluid, modulating inflammatory responses and ECM degradation [81]. H19 interacts with miR-140-5p to regulate cartilage matrix degradation and calcification, and it sponges miR-106a-5p to influence chondrocyte proliferation and apoptosis under IL-1β stimulation [82,83]. Additionally, H19 upregulates IL-38, which binds to the IL-36 receptor, thereby suppressing inflammation and reducing cartilage damage in OA models [81]. H19 was identified as a key regulator of chondrocyte’s differentiation and endochondral ossification, maintaining the anabolic/catabolic activities of chondrocytes and proliferation, whereas its downregulation seemed to promote the hypertrophic differentiation of chondrocytes, a feature of OA joints [84,85,86]. Moreover, H19, acting as a ‘sponge’ for various miRNAs, can modulate the expression of catabolic enzymes (MMPs and ADAMTSs), proposing that the disrupted expression of H19 could affect the chondrocytes’ proliferation and ECM balance, contributing to OA onset and progression [82,87]. However, in meniscus pathology, the role of H19 is less studied, but its involvement in ECM regulation and inflammatory pathways suggests potential implications in meniscal degeneration.

In multifactorial diseases, where local, systemic and genetic factors interact, the identification of a distinct transcriptional signature in disease-affected tissues, accompanied by in silico approaches, is considered a powerful tool for early-stage research and drug development. Regarding OA, MMP-13 and RUNX2, master OA-related molecules that trigger cartilage degradation are considered key targets for developing a novel OA therapy [88,89]. Molecular modifications that affect the activity of MMP-13 or RUNX2 could change the abnormal function not only of cartilage but also meniscus, facing OA as a whole-joint disease and not as a cartilage disease. Moreover, a specific gene set (*MMP-13*, *RUNX-2*, *ITGB2*, *CD36*, *TBX4*, *GJB2*, *PAQR5*, *CLEC12A*, *lncRNA XIST* and *lncRNA H19*) related to disrupted processes observed during OA, such as extracellular matrix organization, inflammation and cholesterol metabolism, could be used as potential biomarkers for the diagnosis of OA at an early stage or for treatment targets, delaying the progression of disease and improving the quality of life of the OA patients. Among the above genes, MMP-13 and ITGB2 could be targeted by known drugs (small molecules), making them potential targets for intra-articular intervention. Moreover, CD36, a scavenger receptor involved in lipid metabolism and inflammation as well as in chondrocyte hypertrophy [90], can be modulated by compounds such as sulfosuccinimidyl oleate (SSO) [91,92], whereas monoclonal antibodies targeting *CLEC12A* are under investigation in autoimmune and inflammatory disorders [72,93], suggesting that key genes in the OA meniscus transcriptional network could represent promising targets for OA. While drug development mainly focuses on disease-associated proteins, RNA has recently been shown to be druggable for therapeutic purposes as well. Small molecules that target the functional transcriptome, including lncRNAs, are a promising therapeutic strategy for complex diseases including OA. Regarding lncRNA XIST, Nickbarg et al. revealed that a small-molecule drug was capable of disrupting the structure and function of XIST in both in vitro and in vivo experiments [94]. However, to date, there has been little progress in the development of small-molecule drugs against lncRNAs, and no lncRNA-targeting therapies have been approved.

Several potential limitations of our study should be addressed in future work. First, the analysis was based on a relatively small cohort, which may have reduced the statistical power and limited the generalizability of our findings. Larger-scale transcriptomic analyses involving OA and healthy meniscal tissues are necessary to confirm our results. In addition, the lack of detailed age information in some datasets—or substantial age differences between OA and healthy groups—emphasizes the need for future studies using age-matched cohorts to better distinguish OA-related molecular changes from those associated with aging. Another important limitation is that this study was based on solely in silico analysis, and no experimental validation was performed to support the results of differential gene expression analysis and the computationally predicted pathway. Since in silico analyses can oversimplify biological systems and may not fully capture the complexity of multifactorial diseases like osteoarthritis, experimental validation through in vitro and in vivo studies is essential. Functional studies, including loss- or gain-of-function experiments, are needed to clarify the functional implications of transcriptomic changes in meniscal degeneration.

In conclusion, our results demonstrated distinct transcriptomic changes in the OA meniscus, affecting biological processes and pathways related to OA onset and progression. Key molecular drivers of cartilage degradation, MMP-13, RUNX2, ITGB2, lncRNA XIST and lncRNA H19, were also implicated in meniscal degeneration associated with OA, highlighting them as suitable candidate targets for developing new drugs that can modulate the function of all joint tissues affected by OA. Notably, we also identified three novel overlapping genes (*GJB2*, *PAQR5* and *CLEC12A*) as being differentially expressed among meniscus, cartilage and synovium OA tissues that had not been correlated with OA. As predicted, their role in OA-related biological processes makes them novel targets for future OA therapy.

## 4. Materials and Methods

### 4.1. Datasets

The inclusion criteria for the selection of datasets used in our study were as follows: (i) human samples, (ii) publicly available RNA-seq datasets and (iii) a sample size ≥ 3. The exclusion criteria were as follows: (i) animal samples, (ii) datasets generated using microarray platforms and (iii) datasets involving treated cells. The dataset GSE185064 that included RNA-sequencing (RNA-seq) data was downloaded from the GPL24676 platform (Illumina NovaSeq 6000, Illumina, Inc., San Diego, CA, USA, *Homo sapiens*) and included 4 OA meniscus samples and 4 healthy meniscus samples (1 woman and 3 men; mean age 38.75 years, range 18–50 years). Healthy meniscus samples were collected from patients who underwent amputation and did not have OA or rheumatoid arthritis, whereas the Kellgren–Lawrence grading score was 0. No detailed information about the gender and mean age of OA patients was included [23]. The dataset GSE114007 that included the RNA-seq of 18 healthy (5 women and 13 men; mean age 38 years, range 18–61 years) and 20 OA (12 women and 18 men; mean age 66 years, range 52–82 years) human knee cartilage tissues [95], was downloaded from the GPL11154 (Illumina, HiSeq 2000, Illumina, Inc., San Diego, CA, USA, *Homo sapiens*) and GPL18573 (Illumina NextSeq 500, Illumina, Inc., San Diego, CA, USA, *Homo sapiens*) platforms. The dataset GSE143514 that included the mRNA profiles of the synovial tissues of 5 OA patients and 3 normal controls were obtained from GPL20795 (Illumina, HiSeq X Ten, Illumina, Inc., San Diego, CA, USA, *Homo sapiens*). The search results did not provide specific information about the gender and mean age of the sample donors in the GSE143514 dataset [26]. All the above-mentioned datasets were obtained from the Gene Expression Omnibus (GEO) database (https://www.ncbi.nlm.nih.gov/geo/ (accessed on 10 January 2025)). Table 4 summarizes the information of each dataset used.

### 4.2. Differential Gene Expression Analysis

Differentially expressed genes (DEGs) were obtained using R (version 4.4.2) and the ‘DESeq2’ package [96]. The adjusted *p*-value (padj) < 0.05 and absolute log2 fold change (log2FC) > 1 were set as the chosen threshold values to identify significantly differentially expressed genes to achieve both high sensitivity and specificity in identifying biologically significant changes. Volcano plots of DEGs and heatmap graphs were generated via the ‘ggplot2’ package [97] to display the results of differential expression analysis.

### 4.3. Construction of Protein–Protein Interaction (PPI) Network

The Search Tool for the Retrieval of Interacting Genes/Proteins (STRING) database (https://string-db.org/ (accessed on 7 February 2025)) was used to generate a Protein–Protein Interaction (PPI) network with the DEGs and an interaction score of 0.4 was set as the threshold [98]. The PPI network was visualized using Cytoscape (version 3.10.3) (https://cytoscape.org/ (accessed on 10 February 2025)), where each node represented a gene or protein, and the edges between nodes indicated their interactions. Clustering of the network was performed using the Markov Cluster Algorithm (MCL) via the ClusterMaker2 plugin in Cytoscape. The inflation parameter was set to 2.7 to control cluster granularity and edge weights were derived from STRING. The remaining parameters were set to their default values with the undirected graph assumption, a maximum residual of 0.001 and a number of iterations of 16.

### 4.4. Enrichment Analysis

Database for Annotation, Visualization and Integrated Discovery (DAVID): Functional Annotation Tool (https://davidbioinformatics.nih.gov/summary.jsp (accessed on 29 March 2025)) was used to perform Gene Ontology (GO) and Kyoto Encyclopedia of Genes and Genomes (KEGG) pathway enrichment analyses of DEGs [99]. Enrichment analyses of the clustered genes were performed with the ClueGo plugin of Cytoscape. Significantly enriched functions or pathways were identified with the *p*-value < 0.05 criterion. Single-sample gene set enrichment analysis (ssGSEA) was performed using the “clusterprofiler” and “msigdbr” R packages [100].

### 4.5. Identification of OA-Related Genes, Transcription Regulators and lncRNA Targets

The genes associated with OA as well as known drugs were selected from the Open Targets platform (https://www.opentargets.org/ (accessed on 5 February 2025)) [101]. Animal Transcription Factor Database (AnimalTFDB) 4.0 (http://bioinfo.life.hust.edu.cn/AnimalTFDB4/ (accessed on 25 November 2024)) was utilized to retrieve transcription regulator genes (transcription factors and cofactors) [102]. Targets of lncRNAs were obtained by the lncRNA2target database (http://123.59.132.21/lncrna2target (accessed on 15 March 2025)) [103]. Venn diagrams were constructed using the Interactive Venn instrument (https://www.interactivenn.net/ (accessed on 8 June 2025)) [104].

## Figures and Tables

**Table 1:** GO and pathway enrichment analysis of differentially expressed genes in OA meniscus.

Description	*p*-Value	Genes
**A. GO Biological Processes**		
Retinol metabolic process	0.001	*CYP2C8*, *RBP4*, *ALDH1A2*, *ADH7*
Ossification	0.006	*IGSF10*, *CORO7-PAM16*, *TWIST1*, *RUNX2*
Embryonic skeletal system development	0.01	*KIAA1217*, *RBP4*, *DLX4*
Bone mineralization	0.03	*MMP13*, *PKDCC*, *RUNX2*
**B. GO Cellular Components**		
Extracellular region	1.14 × 10^−4^	*NGFR*, *PKDCC*, *CPXM2*, *HEG1*, *WNT7B*, *CFI*, *F13A1*, *AGT*, *ADAMTS16*, *IGSF10*, *MFAP4*, *RBP4*, *MMP13*, *GDNF*, *SCG5*, *AMTN*, *FAM180B*, *LCN2*, *CNTN3*, *APOD*, *CD34*, *F2RL2*
Plasma membrane	0.05	*RTN4R*, *VIPR2*, *CLEC12A*, *GPR88*, *CFI*, *ITGB2*, *ADH7*, *RGS1*, *CD36*, *CD34*, *SLC18A2*, *LRRC4B*, *NGFR*, *MCOLN3*, *PAQR5*, *WNT7B*, *TNFRSF10C*, *KCNK15*, *OPRK1*, *SORBS2*, *GFRA3*, *ADRA2B*, *GJB2*, *CYP2C8*, *PLPP4*, *ACKR4*, *CNTN3*, *NECTIN4*, *DSC2*, *F2RL2*
Specific granule membrane	0.05	*CLEC12A*, *ITGB2*, *CD36*
**C. GO Molecular Functions**		
DNA-binding transcription factor activity, RNA polymerase II-specific	0.008	*DLX4*, *IRX5*, *SP140*, *TWIST1*, *ZIC4*, *EVX1*, *KLF14*, *TBX4*, *RUNX2*, *ZNF41*, *ZIC1*, *SHOX*, *SRY*
Cytoskeletal motor activity	0.016	*GPR88*, *KIF5A*, *MYH11*
**D. Pathway Enrichment Analysis**		
PPAR signaling pathway	0.046	*FABP4*, *PLIN1*, *CD36*
Interleukin-4 and interleukin-13 signaling	0.0022	*ITGB2*, *LCN2*, *F13A1*, *TWIST1*, *CD36*
Metabolism of lipids	0.037	*CYP2C8*, *FABP4*, *PNPLA3*, *PLIN1*, *CD36*, *TNFAIP8L2*, *CYP4F11*, *AGT*, *GLB1L3*
Extracellular matrix organization	0.05	*ADAMTS16*, *MFAP4*, *MMP13*, *CAPN6*, *ITGB2*

**Table 2:** Overlapping genes between OA meniscus and synovium tissues.

	Meniscus		Synovium	
**Gene Symbol**	**Log2FC**	**Adj. *p*-Value**	**Log2FC**	**Adj. *p*-Value**
*CORO7-PAM16*	−4.088	0.0149	3.091	9.40 × 10^−9^
*GDF10*	−3.756	0.0397	4.715	0.0003
*GJB2*	3.689	0.0481	7.299	1.09 × 10^−10^
*PAQR5*	4.240	0.0092	3.366	0.0266
*CLEC12A*	6.475	3.45 × 10^−7^	3.729	0.0097

**Table 3:** Overlapping genes between OA meniscus and cartilage tissues.

	Meniscus		Cartilage	
**Gene Symbol**	**Log2FC**	**Adj. *p*-Value**	**Log2FC**	**Adj. *p*-Value**
*TBX4*	−4.973	0.0006	−1.552	1.95 × 10^−6^
*C1orf226*	−4.277	0.0082	−1.166	0.0051
*APOD*	−5.988	4.62 × 10^−6^	−1.646	0.0400
*OPRK1*	−5.090	0.0004	−2.461	0.0170
*GLB1L3*	6.139	0.0040	1.599	4.43 × 10^−5^
*WNT7B*	5.558	0.0220	1.880	0.0063
*AMTN*	5.543	0.0007	4.517	8.24 × 10^−9^
*TNFAIP8L2*	5.482	0.0496	1.446	0.0169
*ALDH1A2*	4.224	3.26 × 10^−8^	1.039	0.0022
*ADAMTS16*	4.178	0.0373	1.303	0.0088
*MMP13*	3.587	0.0091	3.699	2.94 × 10^−12^
*SCG5*	3.310	0.0220	1.285	0.0284
*GDNF*	3.025	0.0095	2.961	0.0002
*CFI*	2.738	0.0096	3.324	5.09 × 10^−29^
*GPR88*	2.356	0.0457	1.134	0.0117
*ITGB2*	2.337	0.0399	3.373	3.14 × 10^−6^
*HEG1*	1.962	0.0220	1.436	9.48 × 10^−7^
*TWIST1*	1.527	0.0352	1.773	1.89 × 10^−5^
*RGS1*	6.919	0.0334	−1.316	1.62 × 10^−6^
*CYP4F11*	6.586	0.0054	−1.375	0.0280
*F13A1*	4.190	0.0034	−1.819	3.41 × 10^−6^
*KIF5A*	−1.969	0.0480	1.521	0.0145
*PKDCC*	−2.290	0.0149	1.565	0.0008
*CNTN3*	−3.167	0.0227	2.232	0.0328
*GJB2*	3.689	0.0481	3.388	7.59 × 10^−6^
*PAQR5*	4.240	0.0092	1.774	0.0210
*CLEC12A*	8.168	3.45 × 10^−7^	2.688	0.0234

**Table 4:** Information of the GEO datasets.

Dataset	Platform	Manufacturer	Group		Tissue
			Normal (Mean Age)	OA (Mean Age)	
GSE186064	GPL24676	Illumina NovaSeq 6000	4 (38.6)	4 (unknown)	meniscus
GSE114007	GPL11154 GPL18573	Illumina HiSeq 2000 Illumina NextSeq 500	18 (38 years)	20 (66 years)	cartilage
GSE143514	GPL20795	Illumina HiSeq X Ten	3 (unknown)	5 (unknown)	synovium

## Data Availability

Data are contained within the article and Supplementary Materials.

## Supplementary Materials

The following supporting information can be downloaded at: https://www.mdpi.com/article/10.3390/ijms26146651/s1.
